# Supplementary material for: Standardized and Quantitative ICG Perfusion Assessment: Feasibility and Reproducibility in a Multicentre Setting
Source: Life (Basel). 2025 Dec 5;15(12):1868. doi: 10.3390/life15121868 (PMC12734919; doi:10.3390/life15121868)
Supplement: Supplementary file 1 [file life-15-01868-s001.zip › Supplementary information A Static example of parathyroid angiography.pdf]

Supplementary materials A – Example fluorescence analysis

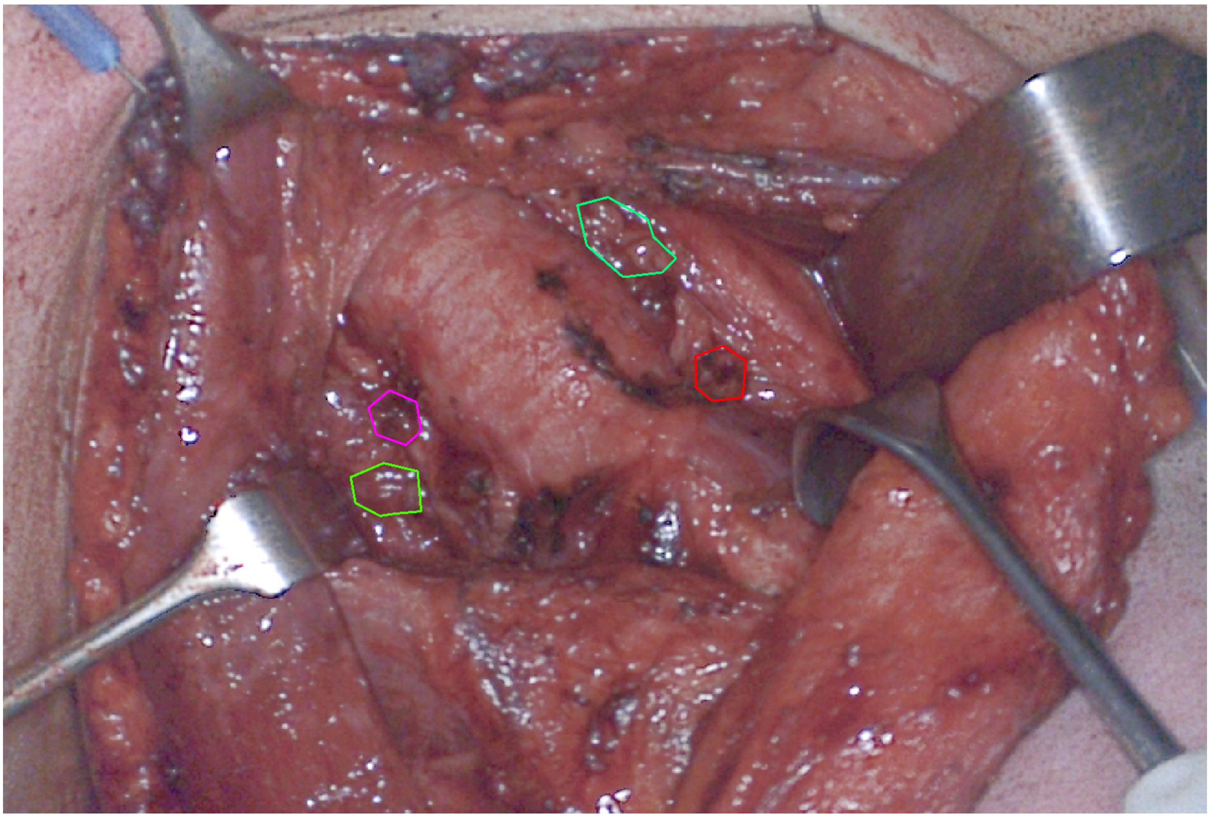

Figure 1. ROI placement around the parathyroid gland. Example of manual ROI placement used for fluorescence analysis. At the end of the fluorescence recording, the surgeon identified each parathyroid gland by gently circling it with an instrument and applying slight pressure to outline its contours. This dynamic manoeuvre clearly delineated the gland's borders on the video, providing a reliable reference for subsequent analysis. Using this indication, a tight circular ROI was drawn manually along the visible margins of the gland in the analysis software, ensuring that the ROI closely followed the gland's anatomical borders without including surrounding tissue. Because still images offer considerably less contrast than dynamic fluorescence video, the gland boundaries appear less distinct in this figure than during actual ROI placement.

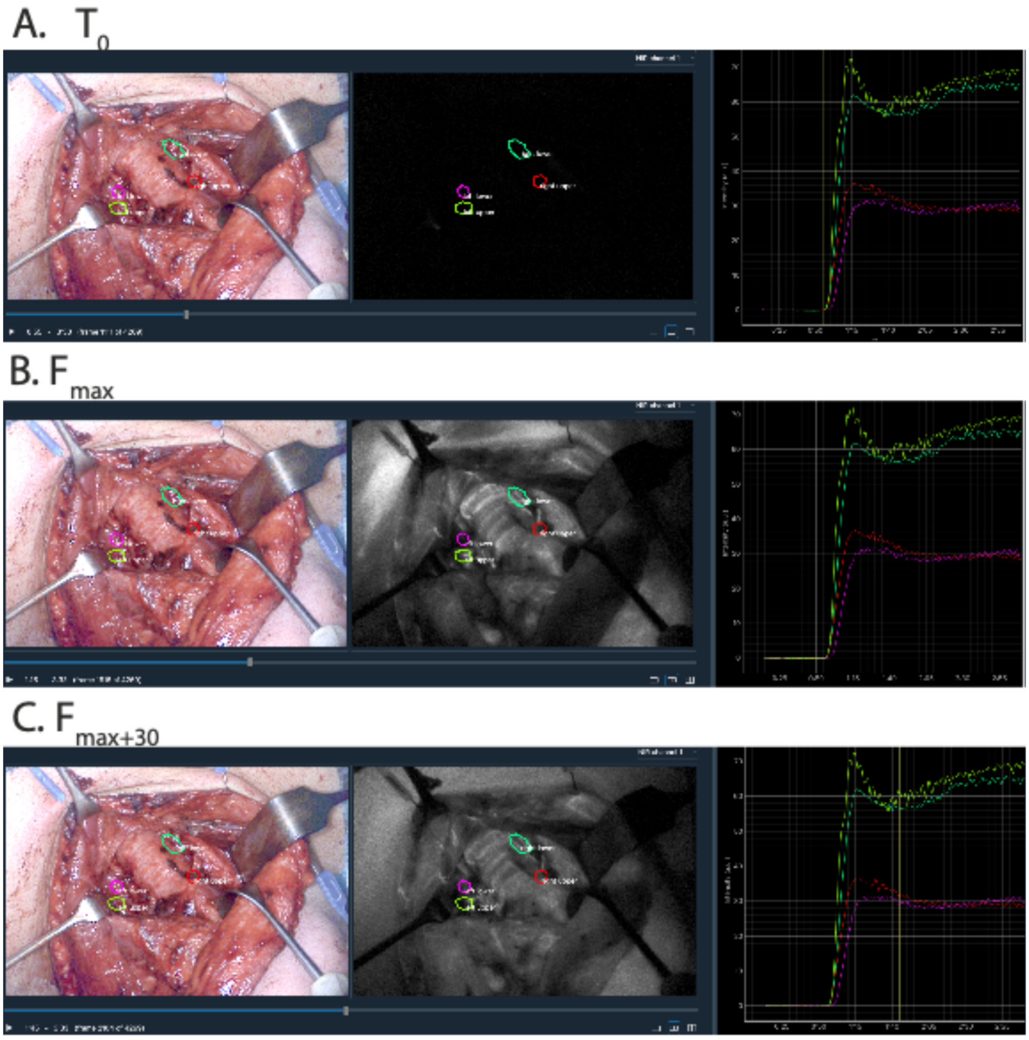

Figure 2. Illustrative case of parathyroid fluorescence angiography presented as static images. Row A (top) depicts, from left to right, time–intensity curves of the parathyroid glands, the corresponding white-light image with delineated glands, and the fluorescence image at baseline (T0). Row B (middle) shows the same sequence at the time of maximum fluorescence (Tmax), and row C (bottom) at 30 seconds after Tmax (Tmax+30s).

Because the parathyroid glands do not appear brighter than the surrounding tissue, visual assessment alone is unreliable. Interpretation therefore requires comparison of each gland with its own dynamic fluorescence course over time, as reflected in the time–intensity curve. It should be noted that this figure provides only a static representation, whereas fluorescence angiography is inherently dynamic.
