# Supplementary material for: Standardized and Quantitative ICG Perfusion Assessment: Feasibility and Reproducibility in a Multicentre Setting
Source: Life (Basel). 2025 Dec 5;15(12):1868. doi: 10.3390/life15121868 (PMC12734919; doi:10.3390/life15121868)
Supplement: Supplementary file 1 [file life-15-01868-s001.zip › Supplementary information B Patient Characteristics.pdf]

Supplementals B – Patient characteristics

Patient characteristics. UMCG = University Medical Center Groningen, EMC = Erasmus Medical Center (Rotterdam), BMI = Body Mass Index, ΔPTH = perioperative decline in Parathyroid Hormone, PG = Parathyroid Gland.

** These patients were excluded because not all parathyroid glands could be assessed due to logistical or video-technical limitations. Further details are provided in Supplementary Information C.*

	Multicenter Cohort			Root Cause Analysis Cohort
	Percentage or median [Q1-Q3]			Percentage or median [Q1-Q3], *n*=15
	**Included; *n*=30**	**Excluded; *n*=35**	***p***	***n*=15**
**Gender: male**	33.3%	42.9%	0.44	13.3%
**Age (years)**	46 [33 – 56]	48 [41 – 61]	0.22	39 [28 – 53]
**BMI (kg/m²)**	27.0 [23.4 – 29.8]	25.5 [22.1 – 29.2]	0.40	27.8 [25.0 – 29.7]
**Indication for surgery**				
• (Suspected) thyroid malignancy (Bethesda 4-6)	26	30		11
• Goiter	1	1		1
• Graves	2	3		2
• Other	1	1		1
**PTH-based HPT (ΔPTH > 80%)**	36.7%	22.9%	0.34	-
**Clinical based HPT (medication)**	3.3%	2.9%		
**Number PGs identified (surgically + pathology)**				
• 1	-	3		3
• 2	-	7		9
• 3	-	19		-
• 4	18	4*		-
• >4	12	2*		-
• N/A	-			3
**≥4 identified PGs per center**				
• UMCG	17			
• EMC	13			
