# Supplementary material for: Standardized and Quantitative ICG Perfusion Assessment: Feasibility and Reproducibility in a Multicentre Setting
Source: Life (Basel). 2025 Dec 5;15(12):1868. doi: 10.3390/life15121868 (PMC12734919; doi:10.3390/life15121868)
Supplement: Supplementary file 1 [file life-15-01868-s001.zip › Supplementary information D All patient curves.pdf]

Supplementary materials D

LIG curves and parameters of all patients included

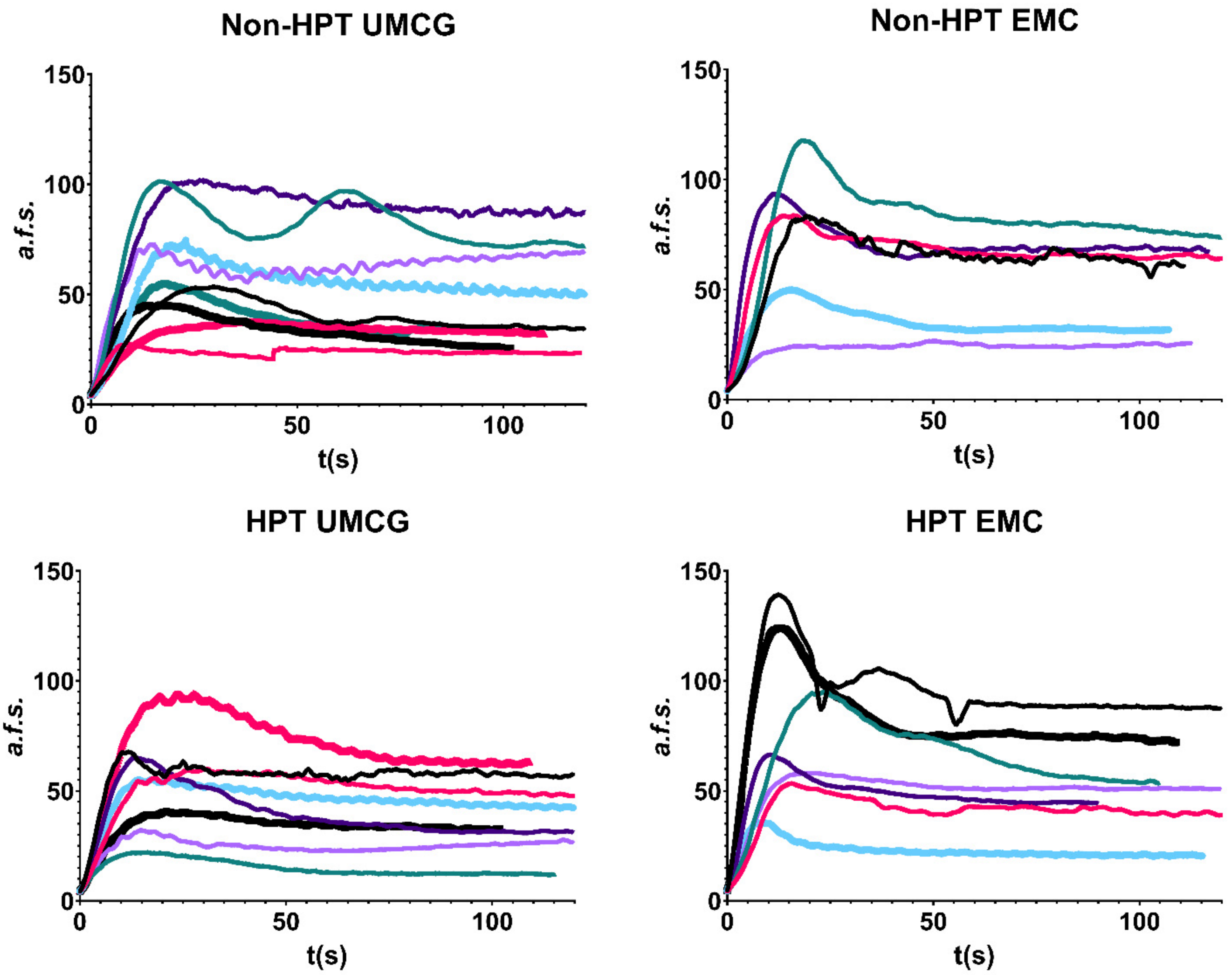

	Non - HPT	HPT	*p*
**F_{max} (a.f.u)**	74 [47 – 94]	59 [43 – 74]	0.59
**Slope 100% (a.f.u./sec)**	3.18 [2.49 – 5.31]	3.39 [1.89 – 5.20]	0.92
**Time to peak (sec)**	14.0 [12.7 – 16.4]	14.0 [10.6 – 18.4]	0.79
**Max Inflow Slope (a.f.u./sec)**	7.63 [4.03 – 10.17]	6.62 [4.41 – 9.61]	0.90
**Outflow Slope 80% (a.f.u./sec)**	-0.80 [-0.92 – -0.55]	-0.49 [-1.15 – -0.22]	0.44

Perfusion parameters of all patients included (UMCG + EMC) using the WISQ model. Patients developing postoperative HPT were compared with non-HPT patients. Values are given as median and interquartile range.
