# Supplementary material for: Standardized and Quantitative ICG Perfusion Assessment: Feasibility and Reproducibility in a Multicentre Setting
Source: Life (Basel). 2025 Dec 5;15(12):1868. doi: 10.3390/life15121868 (PMC12734919; doi:10.3390/life15121868)
Supplement: Supplementary file 1 [file life-15-01868-s001.zip › Supplementary information F Comparison between Quest systems.pdf]

Supplementary information F - comparison between Quest systems

Comparison of Maximum Fluorescence Intensity (F_{max}) Measurements Between EMC and UMCG Systems

To assess the comparability of two Quest Fluorescence Imaging systems at the EMC and UMCG, a detailed agreement analysis was conducted using Bland-Altman methodology, a Q-Q plot, and direct trend visualization across matched measurement values.

The Bland-Altman analysis (Figure 1) demonstrated a mean bias of 4.54 units, indicating that UMCG system consistently reports slightly higher values compared to EMC. The standard deviation of the differences was 7.16 units, with 95% Limits of Agreement ranging from -18.6 to +9.49. No indication of proportional bias was found.

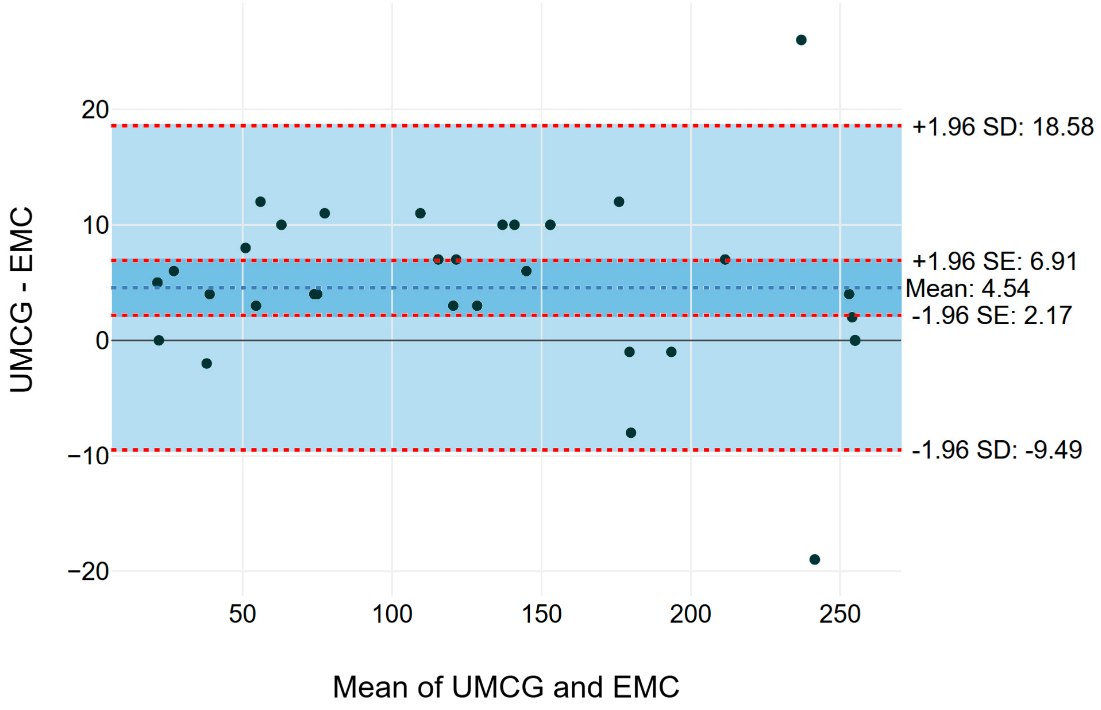

Figure 1. Bland-Altman plot between the camera systems at the EMC and UMCG reporting a mean bias of 4.54 units, indicating that the UMCG system yields slightly higher F_{max} values than the EMC system. However, there is no indication for proportional bias between the systems.

The Q-Q plot (Figure 2) shows that the distribution of the differences approximates a normal distribution, justifying the use of Bland-Altman methodology. This supports the assumption that the differences are not driven by outliers or non-Gaussian features in the data.

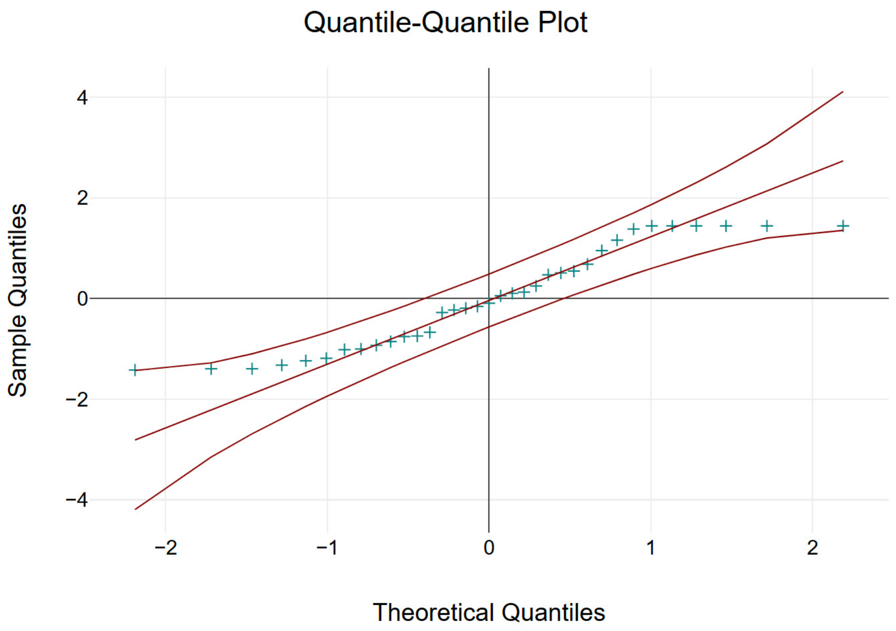

Figure 2. Q-Q plot between the EMC and UMCG camera systems. Although within the boundaries of the 95% Confidence Interval, a slight S-curved deviation was observed. This could be due to minor calibration drifts, sensor variation, variations in light intensity from the system, or environmental differences. Overall, both systems seem to perform rather similar.

The line chart (Figure 3) visually confirms this pattern: both systems follow a similar trajectory in increasing intensity, but UMCG consistently reports slightly higher values. The discrepancy is most impactful in the lower-to-mid intensity ranges, where a fixed difference constitutes a larger proportion of the signal.

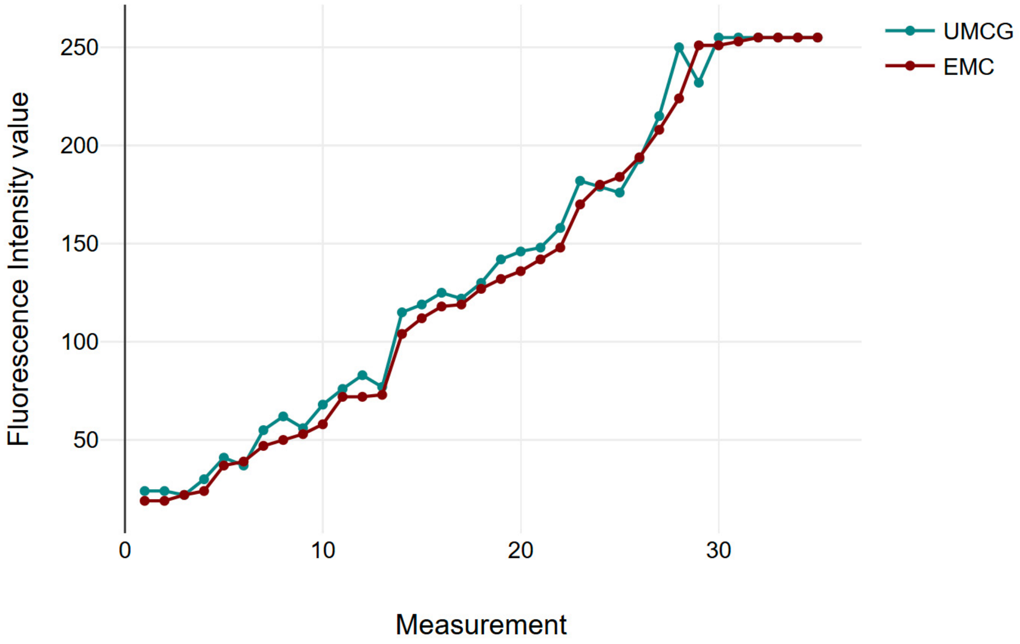

Figure 3. Visual representation of all paired measurements with both systems. Although the system from the UMCG again shows slightly higher values, the trends are very similar.

Conclusion

While there is a slight bias between UMCG and EMC systems, they exhibit high qualitative agreement. For studies relying on precise quantification or threshold-based decisions, a normalization or calibration step is warranted before integrating or comparing data across these platforms.
