# Supplementary material for: Standardized and Quantitative ICG Perfusion Assessment: Feasibility and Reproducibility in a Multicentre Setting
Source: Life (Basel). 2025 Dec 5;15(12):1868. doi: 10.3390/life15121868 (PMC12734919; doi:10.3390/life15121868)

Supplemental G

Skin examples of the root cause analysis cohort

Examples administration rate (skin measurements)

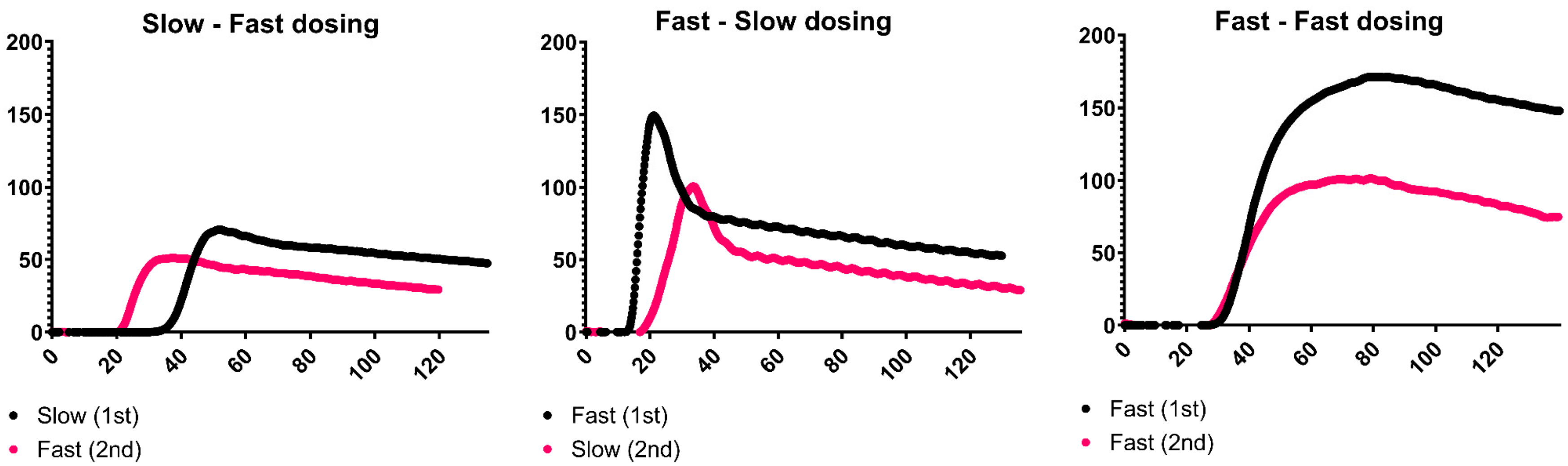

Supplement: Supplementary file 1 [file life-15-01868-s001.zip › Supplementary information G Skin examples of root cause analysis cohort.pdf]
