# Supplementary material for: Standardized and Quantitative ICG Perfusion Assessment: Feasibility and Reproducibility in a Multicentre Setting
Source: Life (Basel). 2025 Dec 5;15(12):1868. doi: 10.3390/life15121868 (PMC12734919; doi:10.3390/life15121868)
Supplement: Supplementary file 1 [file life-15-01868-s001.zip › Supplementary information J Calculations of ICG binding.pdf]

Supplemental J

Calculation concentration Albumin:ICG

- Assuming that blood volume reflects the total distribution volume of ICG, and considering a blood volume of 5000ml, one administration of 7.5mg ICG yields: 7.5/5000 = 0.00150 mg/ml.
- After waiting for 2 minutes for the second administration with a T ½ of 180 s, the following concentration is obtained: $0.00150 + \left(0.001500 \cdot 0{,}5^{\frac{120}{180}}\right) = 0.00244 =$ *0.00244 mg/ml = 0.00244 g/l.*
- *Molecular weight of ICG = 774.96 g/mol.*
- *Molar blood concentration of ICG after the second administration = (0.00000244/774.96) = 3.15*10^-6 mol/L = 3.15 µM.
- In a patient with 45 g/L Albumin, which has a molecular weight of 66.5 kDa = 66500 g/mol, the concentration of albumin is as follows: 45/66500 = 0.676 mM.
- This gives a ratio of Albumin to ICG of 0.676 mM: 3.15 µM.
- **Conclusion:** As expected, there is much more albumin compared to ICG. Additionally, multiple molecules of ICG likely bind to each albumin molecule. Therefore, saturation of albumin does not seem very likely.
