# Supplementary figures and images for: Standardized and Quantitative ICG Perfusion Assessment: Feasibility and Reproducibility in a Multicentre Setting
Source: Life (Basel). 2025 Dec 5;15(12):1868. doi: 10.3390/life15121868 (PMC12734919; doi:10.3390/life15121868)

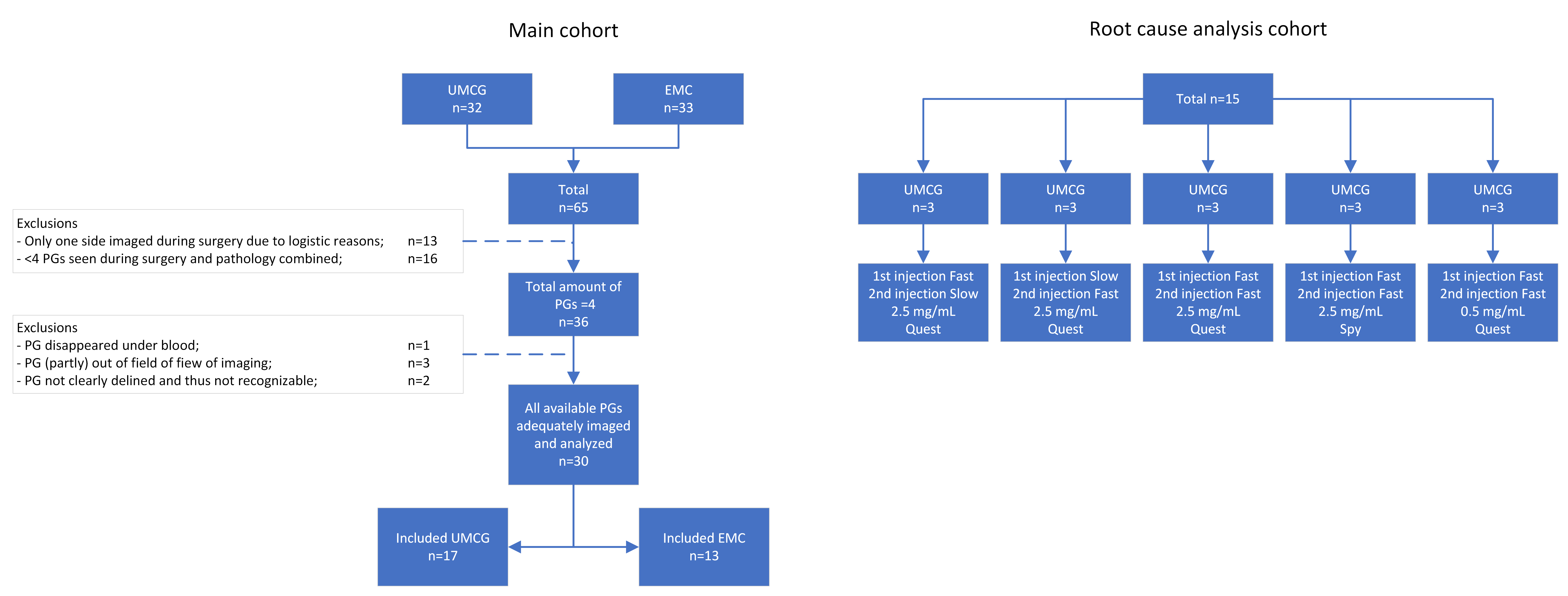

Supplement: Supplementary file 1 [file life-15-01868-s001.zip › Supplementary information C Inclusion and exclusion flowcharts.png]

# Supplementals H

Spy Elite camera results

## Spy Elite data

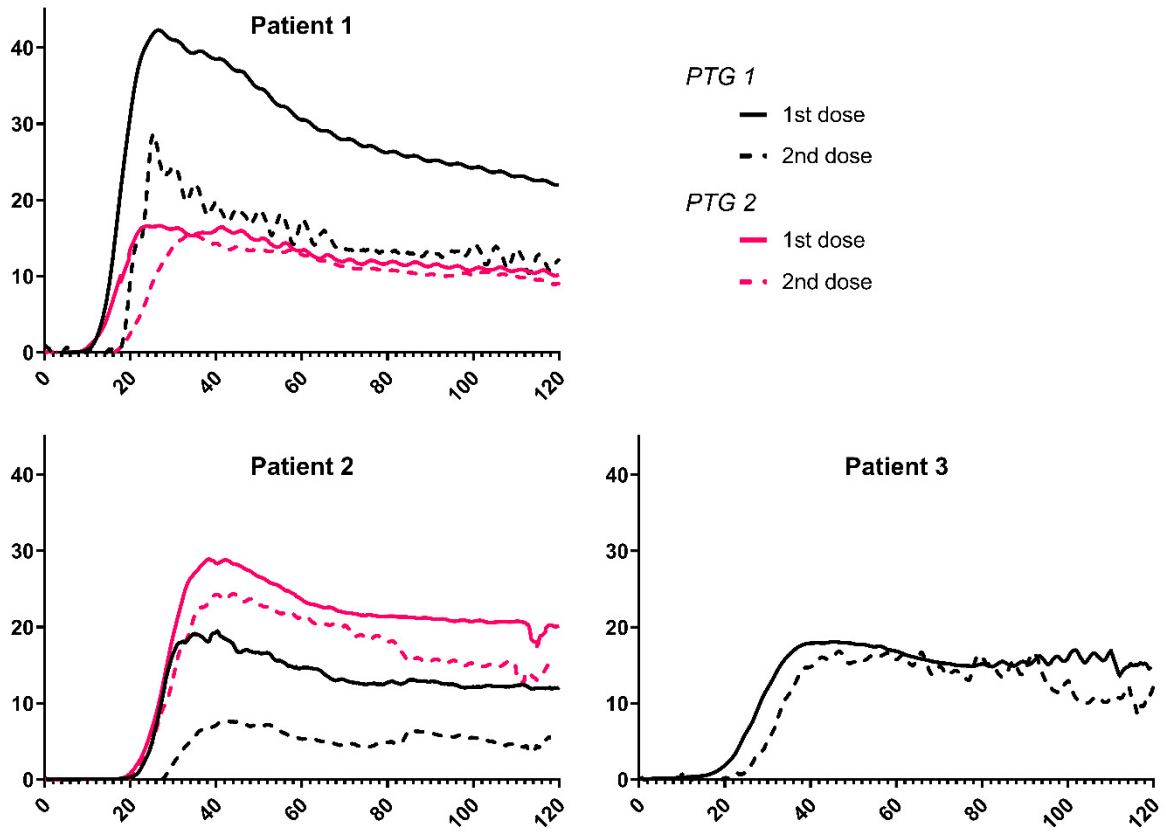

Supplement: Supplementary file 1 [file life-15-01868-s001.zip › Supplementary information H Spy Elite camera results .pdf]
